# Measuring “Where”: A Comparative Analysis of Methods Measuring Spatial Perception

**DOI:** 10.3390/s23239434

**Published:** 2023-11-27

**Authors:** Leah Fostick, Nir Fink

**Affiliations:** 1 Department of Communication Disorders, Auditory Perception Lab in the Name of Laurent Levy, Ariel University, Ariel 40700, Israel; 2 Department of Communication Disorders, Acoustics and Noise Research Lab in the Name of Laurent Levy, Ariel University, Ariel 40700, Israel; nirfi@ariel.ac.il

**Keywords:** sound localization, measuring methods, mean absolute deviation (MAD), root-mean-squared error (RMSE), mirror image reversal errors (MIRE)

## Abstract

The literature offers various methods for measuring sound localization. In this study, we aimed to compare these methods to determine their effectiveness in addressing different research questions by examining the effect sizes obtained from each measure. Data from 150 participants who identified the location of a sound source were analyzed to explore the effects of speaker angle, stimuli, HPD type, and condition (with/without HPD) on sound localization, using six methods for analysis: mean absolute deviation (MAD), root-mean-squared error (RMSE), very large errors (VLE), percentage of errors larger than the average error observed in a group of participants (pMean), percentage of errors larger than half the distance between two consecutive loudspeakers (pHalf), and mirror image reversal errors (MIRE). Results indicated that the MIRE measure was the most sensitive to the effects of speaker angle and HPD type, while the VLE measure was most sensitive to the effect of stimuli type. The condition variable provided the largest effect sizes, with no difference observed between measures. The data suggest that when effect sizes are substantial, all methods are adequate. However, for cases where the effect size is expected to be small, methods that yield larger effect sizes should be considered, considering their alignment with the research question.

## 1. Introduction

Sound localization, a fundamental aspect of auditory perception, is a complex perceptual process that plays a crucial role in our daily lives. It refers to an individual’s ability to perceive and discern the spatial origin of a sound. Sound localization relies mostly on the comparison between the ears of the information perceived. It includes differences in the precise time at which the sound reaches each ear (interaural time difference, ITD) and the intensity of the sound reaching each ear (interaural level difference, ILD) [1]. The ITDs are more effective for localizing low frequencies (lower than 1500 Hz), and the ILDs are more effective for localizing high frequencies (higher than 1500 Hz). Frequencies in the range of 2000–4000 Hz are poorly localized [2,3]. In addition, spectral changes that occur when the sound encounters body parts, such as the torso, head, and pinnae, also provide information on the location of the sound source [1,4].

Appropriate sound localization is important, as it underpins various essential activities that we engage in routinely. It enables us to determine the direction from which a sound emanates relative to our position [3], the distance between us and the sound source [5], and to detect motion [6]. All these enable effective navigation within our surroundings, and, crucially, detect potential hazards. In addition, the ability to localize sound sources enables the selective perception of a specific sound amidst an environment containing a multitude of sounds [5], such as a single speaker at a cocktail party [7]. Given its paramount importance, sound localization has become the subject of extensive research across diverse populations, conditions, and stimuli.

Populations studied in the context of sound localization include mainly those with hearing impairments [8,9,10]. These studies show how individuals with hearing impairments perceive and adapt to auditory spatial information and provide valuable insights into the mechanisms and compensatory strategies they employ to navigate their acoustic environment. An additional way to explore the effect of hearing ability on sound localization is by studying typically hearing listeners who use hearing protection devices (HPDs) (e.g., [4,10,11,12,13,14,15,16,17]). HPDs attenuate the sound’s intensity, causing it to be less clear and distinctive, with a detrimental effect on the localization of the sound source [4,7,8,9,10,11,12,13,14,15,16,17]. Additionally, HPDs that cover the pinnae (e.g., earmuffs) reduce the spectral cues crucial for localizing sound sources placed in front of or behind the listener [4,12,14].

Furthermore, studies of sound localization extend to the types of auditory stimuli employed. As mentioned earlier, the sound’s intensity contributes to its localization by making it clear and distinctive. Its duration affects localization accuracy by providing more (or less) temporal information and energy [14,18]. In addition, sounds longer than ~1.5 s enable the listener to turn the head in the direction of the sound, thereby improving their localization [1,14,19]. The sound’s spectrum also affects the ability to localize it. In general, low-frequency sounds are localized more accurately than those with high frequencies [3,20], although high frequencies facilitate the localization of sounds presented from the back [19,21]. Broad-band sounds are easier to localize than narrow-band sounds, and narrow-band sounds are easier to localize than pure tones [3,13,22,23,24,25,26,27].

The data reported in sound localization studies are collected and analyzed using various methods. These methods compare the participant’s report on the location of the sound source to its actual position. These measures can be divided into four approaches. One group of methods focuses on the gap between the angle of the sound source to the one reported by the listener. The most straightforward measure in this group calculates the difference between the azimuth of the sound source and the position reported by the listener, expressing the response in terms of the mean absolute error (mABSe) [28]. The measure of root-mean-square error (RMSE) manipulates the absolute error, giving more weight to relatively larger errors than small ones [8,15,22,23]. It is a widely used measure, with a small RMSE pointing to localization precision. A second group of methods presents the percentage of errors relative to a defined degree. Such methods are the very large errors (VLE), calculated as the percentage of errors larger than 45° [15]; the percent of errors larger than the average error observed in a group of participants (pMean); and the percentage of errors larger than half the distance between two consecutive loudspeakers (pHalf). A third group considers the directions within a sound field. It can be focused on confusion between close azimuths (such as between sound sources of 30° and 60°) or distant azimuths (such as between 0° and 180°) [13]. A common way to consider localization relative to the sound involves mirror image reversal errors (MIRE)—responses identifying the sound source as coming from the opposite direction than it was delivered [11,12,22]. Lastly, sound localization can be expressed as accuracy—the percentage of responses in which the sound source was accurately identified [29,30]. This measure can be used only when participants know where the sound sources are located (such as with the response box showing the positions of the loudspeakers).

These measurements were recently reviewed by Mertens and colleagues [31], who aimed to propose a standardized framework for comparing sound localization abilities between cochlear implant users and individuals with normal hearing. To further explore the methods used to measure sound localization, the present study aimed to build upon the comprehensive analysis conducted by Mertens and colleagues and determine the effectiveness of different measurements in addressing various research questions. To achieve this goal, we examined the effect sizes obtained from each measure, which were used to address research questions related to differences in sound localization concerning speaker angle (22.5°, 67.5°, 112.5°, 157.5°, 202.5°, 247.5°, 292.5°, and 337.5°), stimuli type (pink noise, spoken word, gunshots), the type of HPD utilized, and the condition (with or without HPD).

## 2. Materials and Methods

### 2.1. Participants

The research study involved a cohort of 150 undergraduate students, whose ages ranged from 20 to 35 years. Among the participants, 55% were females. All individuals underwent a screening process designed to confirm their normal hearing abilities. This assessment included the evaluation of hearing thresholds, which needed to be less than or equal to 25 dB HL across various frequencies (i.e., 500, 1000, 2000, 3000, 4000, 6000, and 8000 Hz). Exclusion criteria included a diagnosis of learning disability or attention deficit hyperactivity disorder, due to their association with a deficit in temporal processing [32,33]. The eligible participants were randomly allocated into five distinct groups. Each of these groups consisted of 30 participants and were tested with a different HPD. 

### 2.2. Stimuli, Apparatus, and Setting

**Figure 1:**
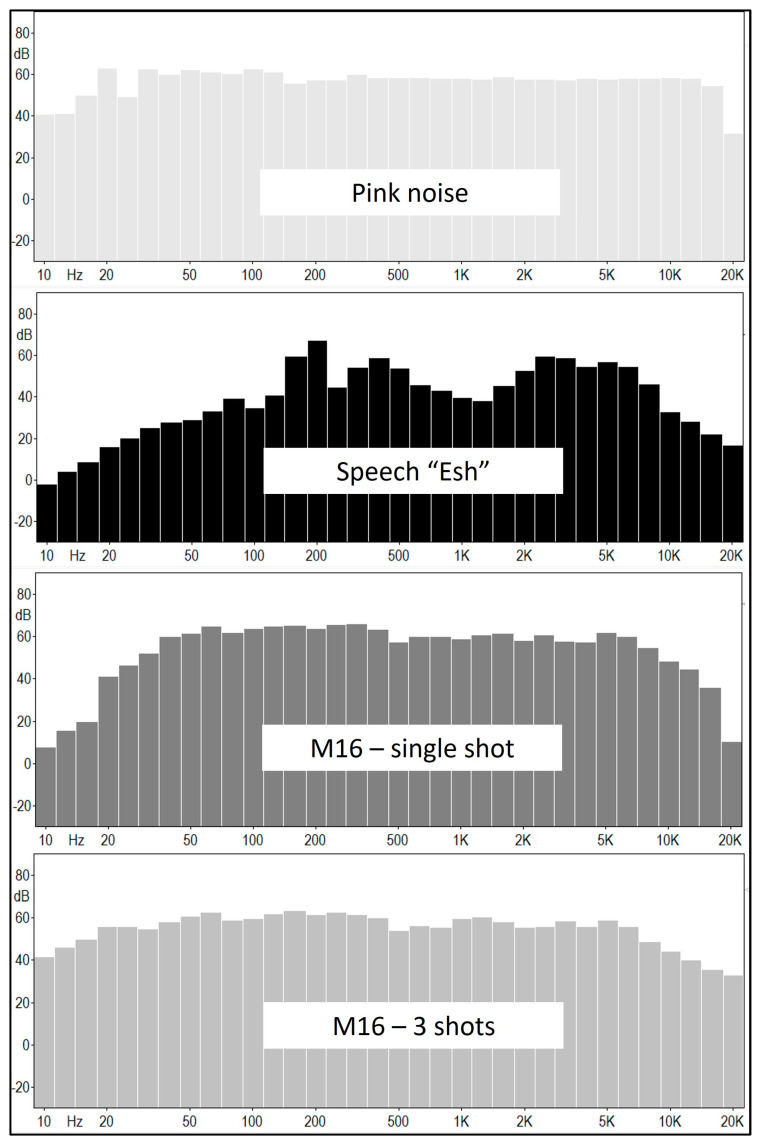
The spectrum of the four stimuli used in the study: pink noise, a spoken word (Hebrew word “esh” meaning fire), and single and triple M16 gunshots captured at a distance of 200 feet.

The data analyzed in this study were gathered using the same stimuli utilized in previous works by Fostick and Fink [22] and Fink et al. [23], and the data collection employed the same equipment and setup. Four stimuli were used: continuous pink noise, lasting 212 ms; a spoken word “esh” (fire) spoken by a male speaker, lasting 409 ms; an impulse noise of a single gunshot from an M16 assault rifle, lasting 202 ms with an additional reverberating tail of 800 ms; and three consecutive gunshots, lasting 660 ms, with an additional reverberating tail of 660 ms. The word was recorded in a sound-treated booth, using an Electro-Voice™ RE320 microphone (Burnsville, MN, USA) connected to a Focusrite™ Saffire Pro™ 24 DSP sound card (High Wycombe, UK), with a Hewlett Packard^®^ computer (Spring, TX, USA) running MAGIX^®^ Samplitude^®^ Pro X 8 software (Hangzhou, China). Pink noise was generated using the Sound Forge™ Pro version 11 software of MAGIX^®^. The M16 assault rifle shot was recorded by Sintonizar Productions [34]. These gunshots were recorded at a distance of 200 feet from the shooter. All stimuli were presented at a sound pressure level of 65 dB SPL. Figure 1 displays the spectrum of each stimulus, as recorded using a GRAS 45CB HATS (Holte, Denmark).

**Figure 2:**
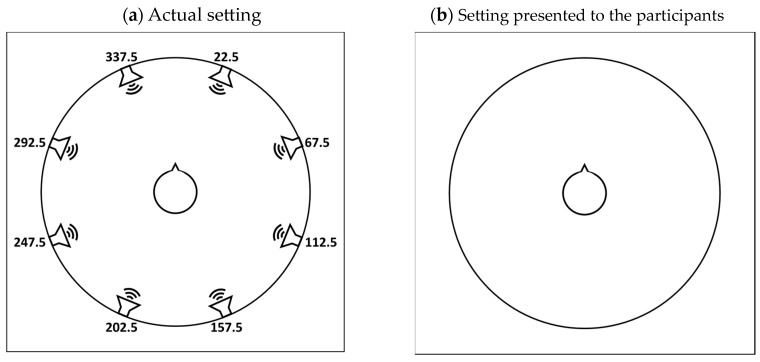
Experimental setting. The symbol in the middle represents participant orientation. (**a**) The actual setting: Participants sat in the middle of a circle of eight monitors separated by 45°, starting from 22.5° through 337.5°. (**b**) The setting presented to participants on a computer screen: participants were asked to indicate on the circle the location of the perceived sound source.

The presentation of auditory stimuli was executed through the utilization of a Steinberg UR824 USB 2.0 Audio Interface™ (Hamburg, Germany), which was integrated into the experimental setup. This audio interface facilitated the transmission of auditory signals to a network of eight RCF Ayra 5 active monitors (Reggio Emilia, Italy), positioned 60 cm from participants and placed in a circular configuration separated by 45°. To avoid a ceiling effect in the localization responses of the participants, who were healthy young adults with good hearing, the monitors’ angles started from 22.5° through 337.5° (as visually represented in Figure 2a). The setting was placed within a soundproof anechoic chamber to create an immersive soundscape that enveloped the participants, enabling them to engage in the sound localization task under controlled and precisely calibrated conditions. Prior to the experiment, the setting was calibrated by injecting each monitor separately with a 1 kHz tone and measuring 100 dB SPL with the GRAS™ 45CB ATF (Holte, Denmark). The ATF was seated in the center of the monitors’ circle, and the calibration tone signal from each monitor was recorded with two 1/2″ microphones situated at the end of the ATF’s simulated ear canals. The microphones were connected to a SINUS™ SAMURAI™ sound level meter (Leipzig, Germany) conforming to IEC 60651/IEC 60804/IEC 61672-1, IEC 651, and IEC 804 standards [35,36,37,38,39].

To carry out the experiment, the participants were comfortably seated with a Dell™ Inspiron™ 13 5378 i5 laptop (Dallas, TX, USA) computer positioned on their laps. The tablet’s screen featured a symbolic representation of each participant along with an indication of their orientation within the auditory environment. Importantly, the visual representation was enclosed by a continuous circular border that thoughtfully concealed the precise positions of the audio monitors (depicted in Figure 2b). This careful design element was implemented to ensure that participants remained unaware of the exact locations of the sound sources. The experiment was carried out using designated software that controlled sound delivery and recorded participant responses. The software was written in C# version NET 4.5.2. In the course of the experiment, as each auditory stimulus was presented, participants were asked to indicate their perceived location of the sound source.

### 2.3. Hearing Protection Devices (HPDs)

Five HPD conditions were tested in the current study and included 3M™ Ear Classic, 3M™ Combat Arms™ 4.1 earplugs in an open mode, 3M™ Peltor™ Bull’s Eye™ H515FB flat earmuffs, a double protection condition combining the last two mentioned HPDs, and the Perforated Concave Earplug (pCEP). The pCEP is a newly developed, proof-of-concept passive HPD, consisting of a concave bowl-like rigid structure attached to a commercial roll-down earplug. The pCEP is designed to improve sound localization with minimal compromising of noise attenuation by Fink et al. [23]. During the experiment, the HPDs were fitted for the participants by the experimenter.

### 2.4. Procedure

The study adhered to Good Clinical Practice (GCP) regulations and received approval from the university’s institutional review board. After providing informed consent and undergoing a hearing screening, participants received brief training where all sounds were randomly presented once from each monitor. A break was given after training, just before the experiment commenced. Half of the participants were initially tested with the HPD condition followed by the no-HPD condition, while the other half underwent testing in the reverse order. An additional break was provided between the HPD and non-HPD conditions. Each stimulus was delivered 10 times from each monitor resulting in 240 trials (3 stimuli type × 8 monitors × 10 repetitions) randomly intermixed by the experimental software. After every 48 trials, the participants were offered a short break. The experiment lasted for approximately 20 min for both conditions, with the entire procedure (including screening and training) lasting almost 60 min. Upon completing the task, participants received monetary compensation amounting to 65 USD as remuneration for their time.

### 2.5. Data Analysis

The analysis encompassed six methods described in the introduction, as outlined below:

1. MAD was computed as the difference between the sound source angle and the angle reported by the listener.
2. RMSE calculated the angular distance between the response angle and the target monitor angle.
3. VLE represented the percentage of errors larger than 45°.
4. pMean denoted the percentage of errors larger than the average error calculated across all participants.
5. pHalf indicated the percentage of errors larger than 22.5°, which corresponds to half the distance between two consecutive loudspeakers in the current setup.
6. MIRE measured the percentage of responses that localized the stimuli to the hemifield opposite the target monitors. This included responses of angles 181° to 359° when the target monitors were on 67.5° and 112.5° (Right/Left MIRE), responses of angles 1° to 179° when the target monitors were on 247.5° and 292.5° (Left/Right MIRE), responses of angles 91° to 269° when target monitors were on 337.5° and 22.5° (Front/Back MIRE), and responses of angles 271° to 360° and 0° to 89° when target monitors were on 157.5° and 202.5° (Back/Front MIRE) (Figure 4 in [22]).

Effect sizes were determined using three-way repeated measures ANOVAs for each measure, incorporating speaker angles (22.5°, 67.5°, 112.5°, 157.5°, 202.5°, 247.5°, 292.5°, and 337.5°), stimuli (pink noise, spoken word, single gunshot, and three gunshots), and condition (with/without HPD) as within-subject independent variables and type of HPD (pCEP, Ear Classic, Combat Arms, Peltor, and double protection) as a between-subjects independent variable. Partial Etta-squared (η^2^_p_) values were calculated using IBM SPSS Statistics Version 29, as SS_effect_/(SS_effect_ + SS_error_) for each main effect, whereas SS_effect_ is the sum of squares of an effect for an independent variable and SS_error_ is the sum of squares error in the ANOVA model. These effect sizes were compared among the different measures.

## 3. Results

### 3.1. Main Effects

In the present study, all main effects were found to be significant, indicating that all methods detected differences in localization between speaker angles, stimuli, type of HPD, and condition. Table 1 shows the main effect and effect size of all independent variables according to each method. As the primary focus of the study was to compare methods, no interactions are currently reported, and no post hoc analyses were conducted.

### 3.2. Effect Size Comparisons

**Figure 3:**
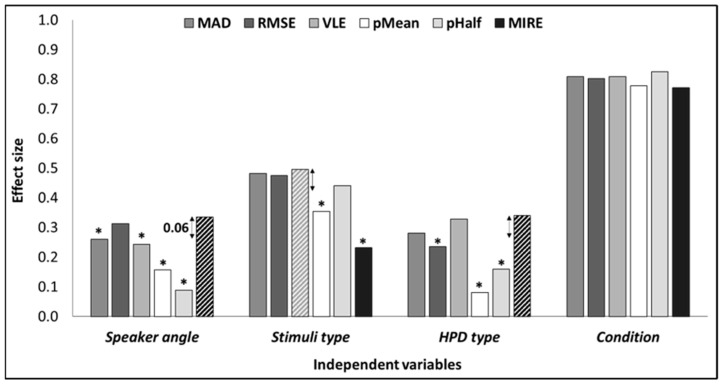
Effect sizes obtained by MAD, RMSE, VLE, pMean, pHalf, and MIRE, for the independent variables speaker angle, stimuli type, HPD type, and condition. The striped bars indicate the measure with the largest effect size for each independent variable. Two-sided arrows indicate the 0.06 distance criteria from the largest effect size. * Effect size significantly smaller (i.e., difference larger than 0.06) than the largest.

Figure 3 presents the effect sizes obtained by MAD, RMSE, VLE, pMean, pHalf, and MIRE, for the independent variables *speaker angle*, *stimuli type*, *HPD type*, and *condition*. The comparison of the effect sizes obtained from the different methods for each independent variable was calculated using a criterion of 0.06, which is considered a medium effect size [40,41]. For each independent variable, the method showing the effect size with the largest value was compared to the other methods. Methods whose effect size was smaller than 0.06, were regarded as *significantly* smaller. For the independent variable *speaker angle*, MIRE yielded the largest effect size (η^2^_p_ = 0.335), significantly greater than the effect sizes obtained from MAD (η^2^_p_ = 0.26), VLE (η^2^_p_ = 0.243), pMean (η^2^_p_ = 0.157), and pHalf (η^2^_p_ = 0.089). The effect size of RMSE (η^2^_p_ = 0.313) was similar to the effect size of MIRE and was also significantly greater than the effect sizes of MAD, VLE, pMean, and pHalf. For *stimuli type*, VLE showed the largest effect size (η^2^_p_ = 0.496), similar to the effect size of MAD (η^2^_p_ = 0.481), RMSE (η^2^_p_ = 0.475), and pHalf (η^2^_p_ = 0.44). The effect sizes of these methods were significantly larger than pMean (η^2^_p_ = 0.354) and MIRE (η^2^_p_ = 0.232). Regarding the *type of HPD*, MIRE obtained the largest effect size (η^2^_p_ = 0.340) and VLE had a similar effect size (η^2^_p_ = 0.328). These methods had significantly larger effect sizes than RMSE (η^2^_p_ = 0.236), pMean (η^2^_p_ = 0.081), and pHalf (η^2^_p_ = 0.16). MAD had a similar effect size (η^2^_p_ = 0.281) as MIRE and VLE but was larger only than the effect sizes of pMean and pHhalf (and not from RMSE). Lastly, the *condition* variable displayed large effect sizes across all independent variables for all measures, with no significant difference observed between measures.

### 3.3. Confidence Interval Comparisons

**Figure 4:**
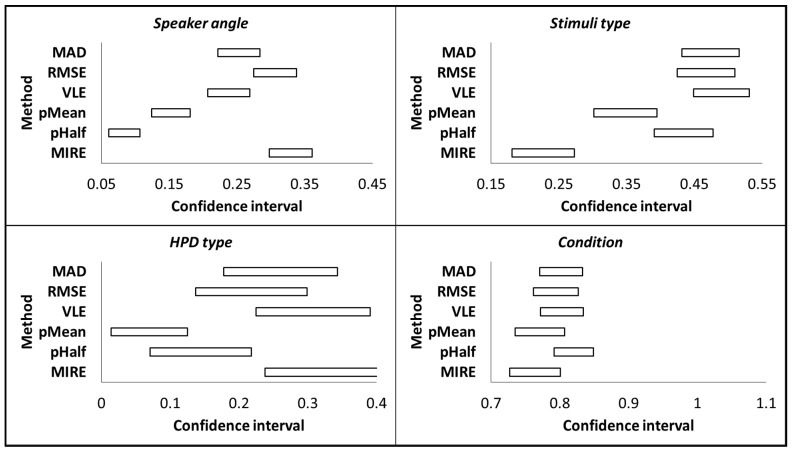
Confidence intervals of the methods MAD, RMSE, VLE, pMean, pHalf, and MIRE for the independent variables *speaker angle*, *stimuli type*, *HPD type*, and *condition*.

To further calculate the differences between the effect sizes of each method, we calculated the confidence interval for each effect size using a procedure suggested by [42]. This procedure calculates the effect sizes of the extreme upper and lower bounds of the mean differences and thus produces CI for the effect size. Given the current research goal to statistically compare between effect sizes, we used CIs that are equivalent to significance testing (*p* = 0.05). Significant results can be achieved if the CIs of two effects do not overlap. To mathematically match the non-overlapping of the CIs to the significance test criteria of *p* < 0.05, the corresponding CIs value was 83.4% [43]. Methods were considered significantly different from each other when their confidence intervals did not overlap with each other. Figure 4 presents the confidence intervals obtained for each independent variable from all methods. The results corroborated the findings previously obtained from the comparison based on the 0.06 effect size criterion. The independent variable *speaker angle* showed that the effect sizes of MIRE and RMSE were similar and significantly larger than the other methods. The effect sizes of MAD and VLE were similar and were significantly larger than that of pMean and pHalf, with pMean significantly larger than pHalf. The independent variable *stimuli type* showed similar effect sizes for MAD, RMSE, VLE, and pHalf. The effect sizes of these methods were larger than that of pMean and MIRE, with pMean also larger than MIRE. The independent variable *HPD type* showed similar effect sizes for MAD, RMSE, VLE, and MIRE. The effect sizes of all these methods were larger than that of pMean. The effect sizes of MIRE and VLE were also larger than pHalf. The independent variable *condition* showed an overlap between the confidence intervals of all methods. Consequently, there was no significant difference in the effect sizes between any of the methods.

## 4. Discussion

The objective of this study was to compare the precision of various methods measuring responses for sound localization for different independent variables. The study investigated the effects of *speaker angle*, *stimuli type*, *HPD type*, and *condition* (with or without HPD) on sound localization precision using six methods: MAD, RMSE, VLE, pMEAN, pHalf, and MIRE. The results showed that different methods varied in their ability to detect sound localization across most independent variables. Specifically, MIRE was found sensitive to the effects of *speaker angle* and *HPD type*; RMSE was found sensitive to the effects of *speaker angle* and *stimuli type*; VLE and MAD were found sensitive to the effects of *stimuli type* and *HPD type*; and pHalf was found to be sensitive to the effect of *stimuli type*. No significant differences between methods were found for the *condition* variable, which displayed the largest effect sizes among all variables for all measures.

MIRE’s sensitivity was evident in capturing *speaker angle*- and *HPD-type* effects. Indeed, the MIRE method is designed to focus on the listener’s general awareness of the hemifield [11,12,22]. This awareness is influenced by the position of the speakers (front versus back). The type of HPD used also affects the perception of position, since over-the-ear HPDs limit front-back cues, while in-the-ear devices do not [4,12,22]. On the other hand, MIRE was less sensitive to *stimuli type*, as this perception is not impacted by the position within a hemifield. RMSE demonstrated sensitivity to all variables, but *HPD type*, as it measures precision related to the position of each loudspeaker, rather than general hemifield awareness. Moreover, RMSE assigns a higher weight to larger errors [9], which was shown to enhance sensitivity to localization responses, compared to MAD, a widely used measure in the literature [2,11,12,13]. On the less sensitive end, pMean was found to capture fewer distinctions between conditions. This method assesses localization precision compared to other participants; thus, it may not be sensitive when participants are relatively homogenous.

A key observation from the study is that when the differences between conditions result in large effect sizes, these differences are evident across all measures (for example, the independent variable *condition*), or almost all of them (for example, but to a lesser extent, the independent variable *stimuli type*). Large effect sizes can also be obtained when participants are provided with a closed set of loudspeakers’ location information on their response panels (such as tablets). Access to this information can yield lower error rates and larger effect sizes. However, the generalization to real-life scenarios of such methods is limited since exact sound source locations are typically unavailable. This method was not tested in the present study (as it included only an open-set design) and should be tested in future studies.

Finally, researchers should carefully evaluate the compatibility of the method of measurement with their specific research objectives. In the present study, we tested four research questions (*speaker angle*, *stimuli type*, *HPD type*, and *condition*) out of many more available in sound localization studies. Further research should focus on additional questions, as well as methods that were not included in the present study.

## 5. Conclusions

Selecting a suitable measure depends on the research questions and the expected effect sizes. Recommendations regarding research questions include, in the present study, research questions related to general hemifield awareness and those related to the precision in localizing every sound source (i.e., loudspeaker). For studies measuring hemifield awareness or variables that affect it (such as HPD), MIRE and methods emphasizing the directions within a sound field are preferable. These methods may not be suitable for variables not related to hemifield awareness (such as stimuli type). Studies focusing on precision in regard to every speaker angle are advised to use methods that measure the exact response, such as MAD, RMSE, and VLE. These methods will be less effective for hemifield-awareness-related research questions. pMean is discouraged when participants are relatively homogenous. Since it is based on mean performance, it can be useful to point out participants who deviate from the average responses. 

In terms of effect size, when substantial differences between experimental conditions are anticipated, any measure may suffice. However, for cases with smaller effect sizes, opting for measures that emphasize large errors (such as RMSE) becomes paramount. Measures with low error rates and large effect sizes, such as those designed with closed-set responses, can also be considered, although the generalization to daily life scenarios may be limited. 

## Figures and Tables

**Table 1:** ANOVA main effects and effect sizes (η^2^_p_) of MAD, RMSE, VLE, pMean, pHalf, and MIRE for speaker angles, stimuli, HPD types, and conditions.

Measure	F	df	*p*	η^2^_p_
*Speaker angles*				
MAD	50.538	7, 1008	>0.001	0.260
RMSE	65.516	7, 1008	>0.001	0.313
VLE	46.541	7, 1015	>0.001	0.243
pMean	27.035	7, 1015	>0.001	0.157
pHalf	14.084	7, 1015	>0.001	0.089
MIRE	72.890	7, 1015	>0.001	0.335
*Stimuli type*				
MAD	133.524	3, 432	>0.001	0.481
RMSE	130.219	3, 432	>0.001	0.475
VLE	142.756	3, 435	>0.001	0.496
pMean	79.309	3, 435	>0.001	0.354
pHalf	114.158	3, 435	>0.001	0.440
MIRE	43.771	3, 435	>0.001	0.232
*Type of HPD*				
MAD	14.055	4, 144	>0.001	0.281
RMSE	11.106	4, 144	>0.001	0.236
VLE	17.691	4, 145	>0.001	0.328
pMean	3.184	4, 145	0.015	0.081
pHalf	6.885	4, 145	>0.001	0.160
MIRE	18.712	4, 145	>0.001	0.340
*Condition*				
MAD	610.386	1, 144	>0.001	0.809
RMSE	584.371	1, 144	>0.001	0.802
VLE	616.130	1, 145	>0.001	0.809
pMean	509.298	1, 145	>0.001	0.778
pHalf	688.523	1, 145	>0.001	0.826
MIRE	488.184	1, 145	>0.001	0.771

## Data Availability

The data presented in this study are available on request from the corresponding author. The data are not publicly available due to ongoing analysis of the data.
